# Sex Differences in Children's Motivation and Action Patterns for Climbing as Behavioral Relicts of Ancestral Sexual-Size Dimorphism

**DOI:** 10.1177/14747049251358630

**Published:** 2025-07-15

**Authors:** Richard G. Coss, Victor K. Geisler, Michael Newmann

**Affiliations:** 1 Department of Psychology, University of California, Davis, CA, USA

**Keywords:** australopithecines, children, climbing behavior, evolutionary persistence, relaxed selection, sexual-size dimorphism

## Abstract

Four studies investigated sex differences in children's motivation and action patterns for climbing playground structures and a gymnasium rock wall to assess any influence of ancestral sexual-size dimorphism limiting tree-climbing agility. Study 1 examined yearly incidences of children aged 3 to 13 falling from monkey bars and jungle gyms in a 1985–1989 National Electronic Injury Surveillance System dataset. Injury incidences of 3- to 6-year-old girls were lower than those of same-aged boys with the inverse occurring between ages 7 through 10 (*p* < 0.001). Study 2 determined that, during two recess periods in 13 elementary schools, 3.14% of enrolled girls were climbing playground structures compared with 1.45% of enrolled boys (*p* = 0.021) who were less inclined to climb as they aged. Study 3 showed that 6 to 8 year-old girls climbing alone perched longer (*p* = 0.0004) on 3 jungle gyms in a regional park longer than same-aged boys. Extended perching by girls might reflect their greater desire for surveillance useful historically for assessing danger. For Study 4, video recordings were made of the climbing actions of 28 children 7- to 12- years of age enrolled in an indoor rock-wall climbing class for beginners. Girls exhibited marked climbing differences (*p* = 0.005), with discriminant function analysis classifying 84.6% of girls correctly and 86.7% of boys correctly. While tree climbing was not studied directly, the sex differences shown in these studies indicates that girls are motivated to climb playground structures more than boys and climb rock walls using different action patterns.

## Introduction

One of the premises of evolutionary psychology is that humans retain behavioral relicts from earlier periods of natural selection in the ancestral environment of evolutionary adaptiveness (Bowlby, 1969; Tooby & Cosmides, 1992). In this conception, the sources of natural selection for these relicts are thought to have been relatively consistent until the decline of hunting and gathering with the advent of agriculture. However, there are much earlier contexts for relaxed natural selection with the gradual shift from presumed arboreal foraging and sleeping to more expansive terrestrial foraging promoted by the biomechanical efficiency of longer legs (Rodman & McHenry, 1980: Sailbene and Minetti, 2003). In the current paper, we examine the expression of climbing in children to assess whether sex differences in climbing motivation and action patterns reflect marked changes in sexual-size dimorphism within the human lineage.

### Effects of Relaxed Selection and the Evolutionary Persistence of Behavior

Research questions on whether various nonhuman species can exhibit behavioral relicts under prolonged relaxed selection have been an ongoing topic spanning more than 150 years. For instance, Darwin's (1885, p. 75) speculated that animal domestication provided a context for relaxed selection and the behavioral persistence of ancestral traits. In one example, Darwin discusses a colleague's observations of sled dogs spreading out when crossing thin ice as possibly derived from Arctic wolves hunting prey in similar situations. There is a plethora of more recent examples of animals expressing vestigial behaviors (reviewed by Rayner et al., 2022). For example, some birds and mammals continue to recognize their former predators after migrating to habitats where predators are rare or absent or when climatic changes preclude former predators. Curio (1969) showed that Darwin's finches (*Geospizinae* spp.) colonizing predator-free Galapagos islands continued to recognize their former predators within a multi-thousand-year time frame of relaxed selection (also see Coss, 1999). Similarly, several populations of California ground squirrels (*Otospermophilus beecheyi*) continue to recognize their historical snake predators for a time frame spanning more than 300,000 years of relaxed selection (Coss, 1991, 1993, 1999). In this ecological context, relaxed selection of snake predation was initiated by squirrel populations continuing to occupy cold habitats unsuitable for their exothermic snake predators during fluctuating ice-age conditions. Even longer evolutionary persistence of behavior is documented for host-brood parasitism that extends into the million-year time frame. For example, New World Bohemian waxwings *Bombycilla garrulus* retain the ability to reject all experimentally implanted eggs from their historically nest-parasitizing brown-headed cowbird *Moluthrus ater* (Peer et al., 2011).

### Children's Motivation for Climbing as a Behavioral Relict

One facet promoting the evolutionary persistence of phenotypic traits is the mutation rate influenced by species-generation time. Due to their longevity, humans have the longest generation time among the hominines (see Langergraber et al., 2012, p. 15,717). Compared with common chimpanzees (*Pan troglodytes*), the prolongation of human phenotypic development delays the onset of puberty, allowing for greater experience in shaping brain plasticity in the still-growing cerebral cortex. Within this framework of delayed maturation, the addition of new behavioral capabilities prior to reaching reproductive age was labeled by De Beer (1958) as hypermorphosis when developmental timing in the expression of phylogenetically older traits is maintained (also see Gould, 1977; Shea, 1983). In particular, African lions (*Panthera leo*) exhibit a presumed canalized motivation to climb trees as cubs and juveniles, a property originally useful in adults for refuge and hunting arboreal prey by their smaller-bodied stem-felid ancestor. However as much heavier adults, lions appear to climb trees defensively only when harassed by elephants and Cape buffalo (Makacha & Schaller, 1969). In the same evolutionary context, the desire of young children to climb trees (Gull et al., 2018) might be an evolutionary remnant of the habitual tree climbing activity of juveniles and adults deep within the human lineage. To examine this conjecture, we conducted four studies that investigated sex differences in children's motivation and actions for climbing that might characterize some retention of ancestral tree-climbing abilities that presumably differed in hominin males and females during the Late-Pliocene epoch.

### Morphological Indices of Hominin Niche Partitioning

The idea of sex differences in habitat use was initially expressed by physical anthropologists Susman, Stern & Jungers (1984, p. 149) when they theorized that the East-African hominin *Australopithecus afarensis* might have engaged in niche partitioning or “sexual dinichism” due to sexual-size dimorphism. Their conjecture was based on differences in postcranial morphology inferring limited ranges of limb motion and evidence that males were nearly double the size of females, akin to a gorilla-like level of sexual-size dimorphism (see Plavcan, 1994; Masao et al., 2016). Due to their smaller body size, *Au. afarensis* females were likely to have been more capable tree climbers than the larger males, a property that enabled more expansive arboreal foraging and choice of nighttime refuge from predators. This form of niche segregation was also argued by Doran (1993) based on the sexual-size dimorphism of common chimpanzees and by van Schaik and colleagues (2009, p. 256) who made a similar argument for orangutans in which sexual-size dimorphism reduces competition.

*Au. afarensis*, is a species with body morphology in relative stasis between 3.9 to 3.0 Ma (million years ago) possibly due to stabilizing selection (Ward, 2002; Drapeau et al., 2005). This specific australopith is generally considered to be the precursor of early *Homo* with its morphological transition adaptively underway by 2.8 Ma (Neves et al., 2023; Villmoare et al., 2015) coincident with increasing aridity associated with the onset of global cooling (deMenocal, 2004; Dupont et al., 2005); albeit, there is still disagreement about the phylogenetic origin and earliest appearance of *Homo erectus* (cf. Du et al., 2021; Herries et al. 2020; Kimbel & Villmoare, 2016).

Relaxed selection for routine tree climbing by hominins likely started in the Late Miocene with the advent of facultative bipedality by hominins *Sahelanthropus, Orrorin*, and *Ardipithecu*s (Crompton et al., 2008; Daver et al., 2022; Stamos & Alemseged, 2023). Diminution of pedal grasping, evident in juvenile *Au afarensis* (DeSilva et al., 2018), also characterized by adult footprints (Masao et al., 2016), would have engendered slow vertical tree climbing with a human-like loading of the hip joint and limited ankle inversion for bracing against the bole (DeSilva, 2009). Moreover, the articular surface of the femoral head and talofibular joint of the small *Au. afarensis* female (Lucy) would have arguably allowed more femoral and ankle range motion than that of her male counterpart (Stern & Susman, 1983, p. 299). Nevertheless, there is a contentious issue about the degree of *Au. afarensis* ankle flexibility constraining arboreal behavior (cf. Latimer et al., 1987; Marchi, 2015, p. 138; Stern, 2000; Venkataraman et al., 2013), facilitating greater dorsiflexion for vertical climbing and greater plantarflexion for reaching branches with the feet (Stern & Susman, 1983), but not with the extreme range of motion found in chimpanzees. The long forearms and curved fingers of *Au. afarensis* (Stern & Susman, 1983; Stern, 2000, p. 125) were particularly important for reaching, gripping, and stabilizing body positions while climbing. Smaller-bodied adult females appear to have exhibited a more cranially oriented shoulder-joint cavity, presumably facilitating overhead reaching and suspension (Alemseged et al., 2006; Green & Alemseged, 2012; Lovejoy, et al., 1982). Despite the ambiguity of whether *Au. afarensis* females retained more primitive morphology adapted to climbing trees than males (Larson, 2012), the marked difference in male body mass relative to females would have accounted for a much greater influence on arboreal niche partitioning in which adult-male foraging was restricted to larger weight-bearing branches. Such niche partitioning is likely to have extended further back in time based on the apparent sexual-size dimorphism of *Au. anamensis*, the putative ancestor of *Au. afarensis* (Du et al., 2021; Kimbel et al., 2006; Ward et al., 2020). Moreover, sexual-size dimorphism may have been present in *Nakalipithecus* spp., an early Late Miocene hominine that is phylogenetically near to the lineage of the last common ancestor of humans and chimpanzees (Almécija et al., 2021; Kunimatsu et al., 2016).

The remains of early *Homo* at an Early Pleistocene site in Dmanisi, Georgia, exhibit several postcranial features, such as small body size and a more cranial orientation of the shoulder-joint cavity, both of which are australopith-like (Lordkipanidze et al., 2007). Nevertheless the body proportions of these individuals are more like those of modern humans and their appearance out of Africa indicates the bipedal capacity for long-distance travel. It is reasonable to argue here that tree climbing within the human lineage would have shifted from presumably habitual in the Late Pliocene (Drapeau et al., 2005) into a more resource-dependent facultative degree by the Early Pleistocene. It is evident that sexual-size dimorphism continued to become more human-like as inferred from 1.5 Ma, Kenyan footprints (Villmoare et al., 2019), thus intensifying relaxed selection for emergency tree climbing for antipredator refuge, but not for resource procuring as in the modern-human context (see Ichikawa, 1981; Kraft et al., 2014).

### Sex Differences in Modern Human Body Morphology as Possible Climbing Relicts

The evolutionary persistence of climbing adaptations is still evident in modern humans, but this effect is much less pronounced for skeletal features and more evident in studies of joint laxity. Unlike early *H. erectus* (Plavcan, 2012; Villmoare et al., 2019), sexual-size dimorphism is relatively modest in modern humans and can be modulated by environmental and social factors during ontogeny (Badyaev, 2002; Kanazawa & Novak, 2005).

Sexual differentiation in skeletal growth occurs during relatively late stages of development (Bogin, 1999). Important for the four studies described herein, adolescent boys and girls are virtually identical in their height trajectories prior to age 13 years (Bogin, 1997, p. 64). Nevertheless, boys and girls do differ in their average peak height velocity, which is 11.9 years for girls and 13.9 years for boys (Sherar et al., 2005). Despite this height-velocity difference, the continuity of preadolescent body height provided the context for selecting children 3 to 13 years of age for studying climbing to preclude growth differences in body height confounding any interpretation of sex differences in the climbing results.

The primary difference between boys and girls relevant to historical climbing ability is the greater flexibility of girls (Bala & Katić, 2009; Corbin & Noble, 1980), an advantage in females that continues into adulthood especially in the lower extremities due to ligamentous laxity (Krivickas & Feinberg, 1996). In particular, ankle joint range of motion constrained by tibiocalcaneus coupling linked to achilles tendon compliance (cf. Kato et al., 2005; Sinclair & Taylor, 2014) would be particularly important for slow vertical tree climbing. In general, females exhibit greater range of motion of their ankles than males in all three orthogonal axes, and girls 9 to 13 years of age have markedly greater ankle eversion than boys (Grimston et al., 1993; Kato et al., 2005). A sex difference in the ankle malleolar articular surface has been shown for an adult Japanese sample at the multivariate level (Sacragit & Ikeda, 1995) that might reveal a subtle phylogenetic retention of a historical climbing trait in bony anatomy.

Males and females differ in hip range of motion, with female showing higher ranges of total rotation, total hip motion, internal rotation, and abduction (Svenningsen et al., 1989). In the context of slow vertical climbing of trees both historically and currently (see Ichikawa, 1981, p. 59; Kraft et al., 2014; Venkataraman et al., 2013), a greater hip range of motion would have permitted closer positioning near the bole for ankle dorsiflexion and inversion that would also facilitate forearm clasping. Although males and females do not differ appreciably in the morphology of the shoulder-joint cavity except for larger male size (Churchill et al., 2001; Maier et al., 2022; Merrill et al., 2009); measures of sex differences in shoulder laxity and range of motion are ambiguous due to ontogenetic plasticity (cf. Lombardo & Deaner, 2018; Maier et al., 2022).

### Rationale for Studying Children's Climbing Motivation and Action Patterns

Coss and Charless (2004) presented arguments from a philosophy of science perspective that the verisimilitude of an evolutionarily based core hypothesis is enhanced by testing multiple auxiliary hypotheses with experiments using different methodologies. Derived from the aforementioned fossil record and sex differences in modern-human mobility, the core hypothesis was that early hominins likely exhibited sexual dinichism in their arboreal and terrestrial activities, including choice of nighttime refuge. Such Pliocene-age niche partitioning might be revealed by the evolutionary persistence of children's precocious cognition and action. The choice of studying children's climbing activity described herein was an extension of the following studies of auxiliary hypotheses showing sex differences in children that inductively supported the core hypothesis of historical sexual dinichism: 1) Five to 8 year-old girls balanced more frequently and effectively than same-age boys with their arms out on a balance beam simulating historical tree-branch walking (Coss & Goldthwaite, 1995). 2) Using sequential images of silhouetted trees and pointing, 3 to 4 year-old girls differed from same-age boys in their choice of arboreal refuge from a lion, with girls selecting sites closer to the crown edge with lower weight-bearing branches precluding access by heavier-bodied arboreal predators (Coss & Moore, 2002). This pattern of defensive climbing to avoid leopards is evident in baboons (Busse, 1980). 3) In simulating the ancient pattern of arboreal sleeping in which lighter-bodied hominin females likely nested in trees more than males to avoid predation, 3 to 4 year-old girls differed from same-age boys in their nighttime fear by selecting the location of something scary lurking under the bed (Coss, 2021). 4) This finding in children was replicated in adult rememberances of their nighttime fear (Coss, 2021) and in a cross-national comparison of adult rememberances (Coss & Blozis, 2021). 5) Three to 5 year-old children exhibited a sex difference in their choice of refuge after viewing briefly a realistic leopard model compared with a deer model in a playground setting (Coss & Penkunas, 2016). Together, these studies of preadolescent children contributed to a protective belt as a Lakatosian defense (see Lakatos, 1970; Meehl, 1990, p. 110) inductively surrounding the core hypothesis that historical sexual dinichism can be revealed by children's precocious habitat perception (Coss & Charles, 2004).

Although these previous studies shed light on the core hypothesis, none of them involved the physical action of climbing. To accomplish this objective, exploratory research on age-related climbing injuries (Study 1) provided insight into the formulation of two auxiliary hypotheses predicting that preadolescent girls would climb playground structures more than preadolescent boys (Study 2) and prefer to perch on top of these structures longer than boys (Study 3). Beyond studies of ladder climbing by children discussed below, the aforementioned sex differences in children's range of motion were expected to be expressed in climbing action patterns when inexperienced children climbed a gym-based rock-climbing wall (Study 4). No hypotheses predicting sex differences were formulated for this study, consistent with field studies of unique animal behavior that provide only reliability estimates useful for subsequent hypotheses development. As such, we view this climbing study as exploratory, but useful for evaluating the core hypothesis that behavioral remnants of historical sexual dinichism can be expressed in the precocious climbing behavior of preadolescent children.

## Study 1: Falling Injuries

The U.S. Consumer Product Safety Commission (CPSC) maintains records of hospital emergency room visits involving product-related injuries. For more than 45 years, the CPSC has maintained the National Electronic Injury Surveillance System (NEISS), documenting 130 hospital emergency department (ED) visits throughout the United States (Rivara et al., 1982). Due to playground injuries, most stand-alone climbing structures (i.e., jungle gyms) on elementary school playgrounds and public parks had been replaced by the mid 2000s with typically large integrated structures consisting of platforms with railings, ladders, slides, and attached monkey bars positioned on bark or rubber surfaces (see Frost & Woods, 2015; Howard et al., 2005, p. 1444; Werner, 1982). The subsequent increased safety features of these climbing structures and pliable substrates reduced the frequency of falling injuries reported in a 2001 to 2008 NEISS database (Tuckel et al., 2018).

To obtain records for older playground equipment relevant for evaluating a sex difference in climbing competence at one-year intervals, we selected an archival NEISS dataset from 1985 through 1989 that specifically identified falling injuries from stand-alone metal jungle gyms and monkey bars. As such, the age-determined frequency of climbing injuries could be viewed as a proxy for evaluating sex differences in the propensity of children climbing jungle gyms and monkey bars in a broader developmental context.

## Materials and Methods

### Participants

A contingency table (Table 1) examined 3,755 cases from the 1985 through 1989 NEISS dataset of children 3 through 13 years of age falling from monkey bars and jungle gyms, receiving injuries severe enough to require hospital ED treatment.

**Table 1.** Contingency Table Showing the Number of Children Injured Between 1985 and 1989 While Climbing Playground Structures Between the Ages 3 Through 13.

	Age										
	3	4	5	6	7	8	9	10	11	12	13
Boys:	148	189	290	351	257	217	179	134	83	62	24
Girls:	109	172	260	288	258	200	196	181	91	41	25

## Results

**Figure 1.**
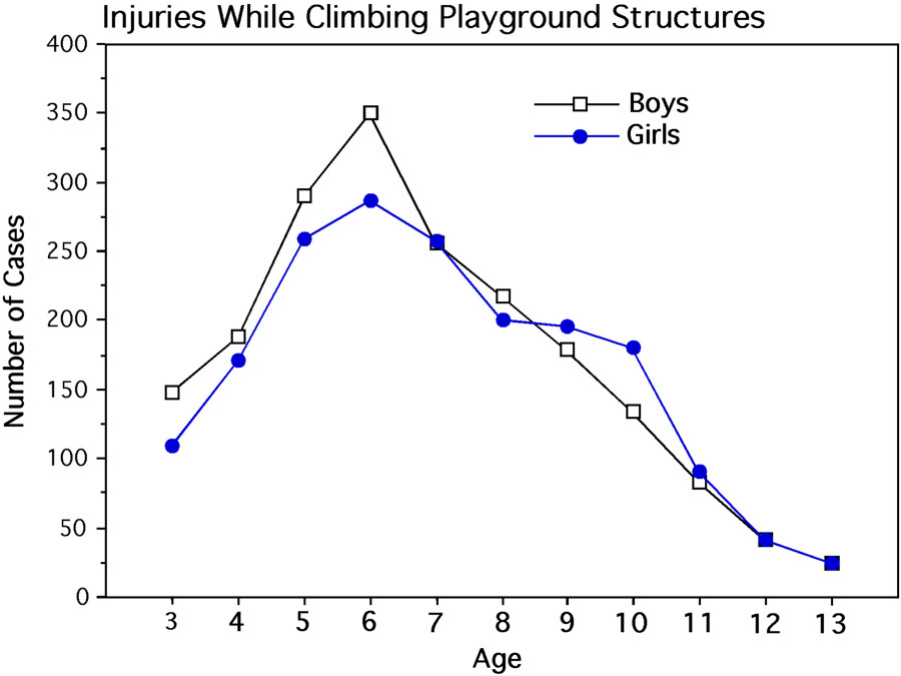
Number of injuries from falling from monkey bars and jungle gyms in the NEISS dataset from 1985 to 1989.

It is apparent from this database (Figure 1) that, prior to age 7, a larger number of boys were injured than girls. However, after age 8, a larger number of girls were injured than boys. Using a single-factor multinomial log-linear analysis with maximum likelihood estimation, the interaction of sex and age was statistically significant (Likelihood Ratio χ^2^_10_ (*N* = 3,755) = 24.405, *p* = 0.007). The major sources of this interaction were identified using data partitioning (Agresti, 2002, p. 82). The number of injuries of 3 through 6 year-old girls were lower than same-aged boys girls with the inverse occurring between ages 7 through 10 (sex by age interaction: likelihood ratio χ^2^_1_ (*n* = 3,429) = 12.794, *p* < 0.001). Partitioned further, this sex difference is apparent in preschool children (age 3) in which more boys experienced climbing injuries than preschool girls using age 7 as the reference point where approximately the same number of boys and girls were injured (interaction of sex and age categories: likelihood ratio χ^2^_1_ (*n* = 772) = 4.072, *p* = 0.044).

## Discussion

The finding that 3 to 6 year-old boys were injured more frequently than same-aged girls suggests that boys engaged in more risk-related action patterns while climbing on jungle gyms and monkey bars. In our NEISS dataset, the incidences of falling in both sexes requiring ED treatment peaked at 6 years of age (Figure 1) in rough agreement with 7.4 years of age for the mean age of falling injuries for children admitted to a single Canadian hospital emergency room after falling from high structures between 1994 and 1997 (Lallier et al., 1999). A more extensive analysis of falling injuries on playgrounds was conducted using the newer Nationwide Emergency Department Sample, the Nationwide Inpatient Sample, and the NEISS database (see Tuckel et al., 2018). Although older age classes including adults were examined, 3 age classes were the primary focus of this particular study, consisting of children less than 5 years of age, 5 to 9 years of age, and 10 to 14 years of age. Across all age classes and types of playground equipment, such as slides, swings, seesaws, and monkey bars, 52.8% of males were injured by falling compared with 47.2% of females, with the majority of patients appearing within the 5 to 9 age class. Among these playground structures, the largest percentage of injuries came from falls from monkey bars that showed a marked decline from 50.5% for children 10 to 14 years of age to 4.8% for juveniles and adults 15 to 24 years of age. This age-related decline in falling injuries is consistent with our findings of an age-related decline in climbing injuries restricted to jungle gyms and monkey bars.

Although climbing is the contextual component of falling, the incidences of children falling from windows one story or higher in New York City from 1965 to 1969 showed a marked decline from 10 to 14 years of age (Bergner et al., 1971). In their analogous study, deaths from falls from heights were consistently higher in boys (69%) than in girls (31%), a property indicating a marked sex difference in a hazardous window-climbing inclination. After the age of 5, girls were also less likely than boys to fall from heights suffering head injuries (Cummins & Potter, 1970).

Developmental changes in the fear of heights that would inhibit playground-equipment climbing might account for the age-related decline in falling injuries. Although some older children do express a fear of heights in survey research (Poulton et al., 1998), there is, to our knowledge, no specific evidence of a sex difference in fear of heights in adolescent children. Furthermore, there is no reliable longitudinal evidence that fear of heights in 11 year olds is affected by their traumatic childhood experiences of falling before age 9, thus negating an experiential conditioning (CS-UCS pairing) conjecture of developmental changes in fear of heights (Poulton et al., 1998). In adults, survey research indicates that women exhibit a higher level of fear of heights than men that reflects a generally consistent trend for many other fears (Fredrikson et al., 1996, p. 36).

Sex difference in the frequency of falling might reflect variation in motor activity engendering greater risks. Other playground studies have shown that boys are generally rated as more active than same-age girls, moving about faster on the ground than girls (e.g., Eaton & Yu, 1989; Ridgers et al., 2006). If translated to a greater pace in arm and leg coordination while climbing, it is reasonable to expect more climbing accidents in boys. In support of faster arm and leg coordination, Butcher (1991) found that 7- to 9-year-old boys were reliably quicker in ladder climbing than same-age girls but there was no evidence of climbing incompetence in either sex. In descending ladders, however, girls exhibited a more efficient leg action pattern than boys did (Kirk, 1989). Such a difference in ladder climbing might be age dependent based on a later Iranian study of ladder climbing of 4- to 6-year-old boys and girls did not differ reliably (Vameghi et al., 2013).

Compared with 7- to 10-year-old girls, the lower injury frequency of same-age boys, shown by the reliable statistical interaction with younger children in our data partitioning, might simply reflect an age-related reduction in climbing motivation by boys due to their emerging interests in other playground activities. Reduction in climbing activity by these older boys would explain their fewer injuries compared with older girls rather than these boys exhibiting an increase in climbing competence. Such a developmental shift in climbing motivation is also present in the decline in tree climbing by juvenile common chimpanzees (Doran, 1992, 1997). Unlike humans, however, these chimpanzees do not exhibit an adolescent growth spurt so that social factors might account for this developmental difference (Hamada & Udono, 2002). Nevertheless, caution should be expressed in interpreting whether the age-related decline in chimpanzee tree-climbing behavior is evolutionarily homologous with that of human climbing due to the long time frame in which these lineages diverged in the late Middle Miocene. To address our speculation about the source of lower climbing injuries in older boys relative to that of older girls, the next study documents the actual climbing frequency observed on elementary school playgrounds.

## Study 2: Climbing Frequency

The falling injuries of boys and girls in Study 1 might simply reflect the frequency of boys and girls climbing jungle gyms and traversing monkey bars. This study examines the frequency of children climbing mostly jungle gyms to help interpret the NEISS injury data. The dataset for this study and that of follow-up Study 3 were generated in the late 1980s and early 1990s well before the elimination of metal jungle gyms on elementary school playgrounds and community parks by the mid 2000s.

## Materials and Methods

### Study Sites

Thirteen elementary schools in the following Northern California cities were sampled to measure the frequency of children's climbing activity: Davis = 4 schools, Dixon = 2 schools, Woodland = 3 schools, Yuba City = 4 schools. These schools were selected for observational study because they all have diverse ethnicities. Permission for observational study of children's climbing was obtained from each school Principal in accordance with the University of California, Davis IRB Human Subjects protocol 91-482R.

### Participants

**Figure 2.**
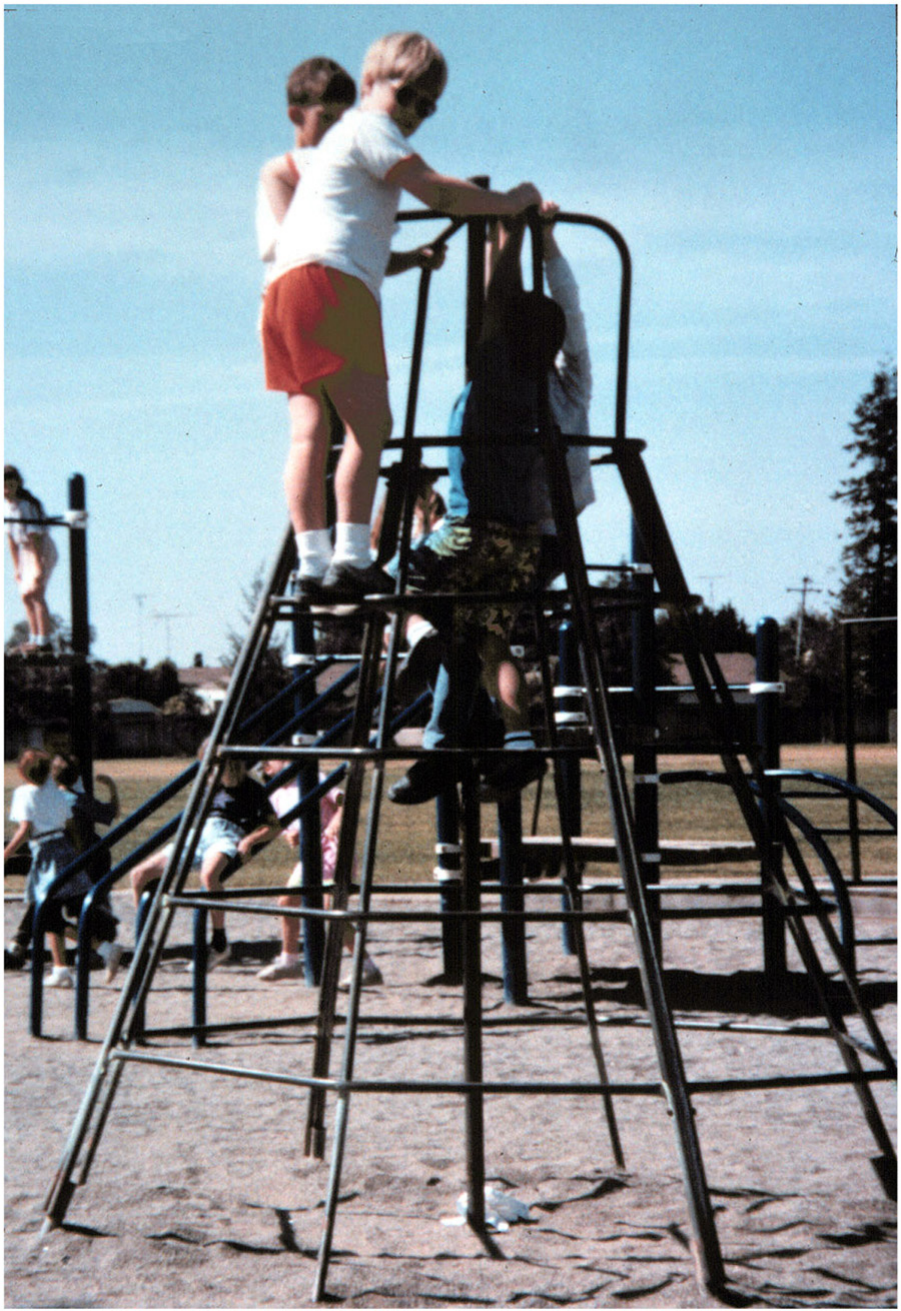
Photograph of children climbing a pyramidal jungle gym during recess.

One hundred and seventy three children (56 boys and 117 girls) were observed climbing playground structures, except rings and swings, during two recess periods that restricted sampling to kindergarten (K) through second grades and third through sixth grades. For sampling reliability, two observers with clipboards counted simultaneously the sex of children on each climbing structure (Figure 2) one time in the same progression of focal sampling of different structures over a 3-min period. Observations were made from outside the playground fences using binoculars. Due to the small numbers of children climbing each structure, no errors of sex identification were found between observers. To avoid a sampling bias, all observers were unaware of the findings of Study 1. The likelihood of resampling the same child on a different climbing structure during our sampling period was very low due to the child's slow descent and selection of another climbing structure.

## Results

**Figure 3.**
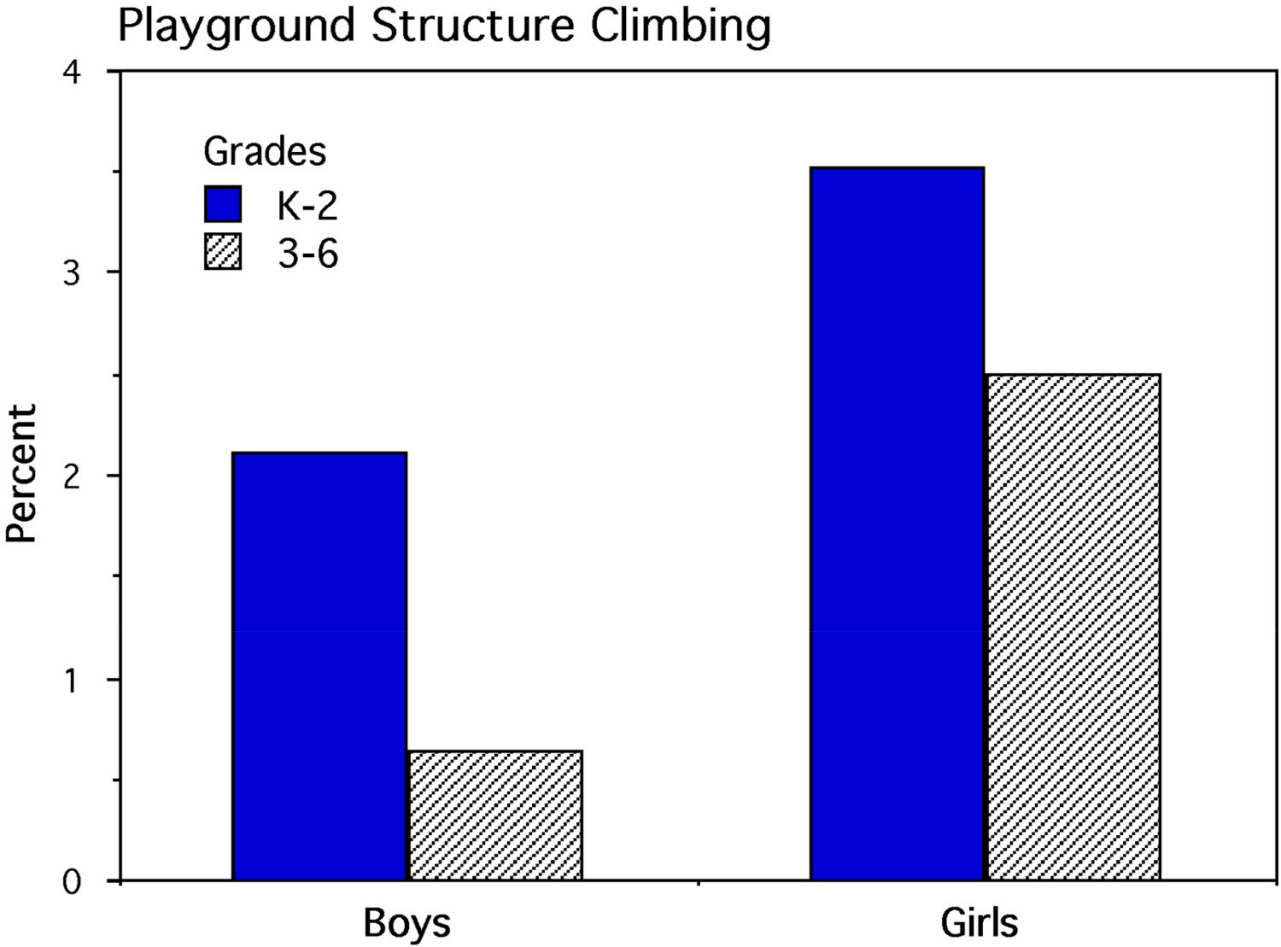
Percentage of children climbing playground structures in 13 elementary schools. The interaction of sex and grade categories is statistically significant (*p* = 0.021).

A contingency table was constructed (Table 2) for analysis by a two-factor (sex and grade categories) multinomial log-linear analysis with maximum likelihood estimation. The number of enrolled children, assuming no school absences, was used to calculate the percentage of children climbing for each grade category (Figure 3). Data partitioning was used to examine planned contrasts for sex and specific grade categories. Analysis showed a major effect for the interaction of sex and all grade categories: likelihood ratio χ^2^_1_ (*N* = 7,759) = 5.366, *p* = 0.021. Contributing to this interaction, the overall percentage of enrolled girls climbing (3.14%) was substantially larger than the overall percentage of enrolled boys who were climbing (1.45%). Data partitioning showed a significant interaction of sex and the K through 2nd-grade category: likelihood ratio χ^2^_1_ (*n* = 4,047) = 7.888, *p* = 0.005, and the interaction of sex and the 3rd- through 6th-grade category: likelihood ratio χ^2^_1_ (*n* = 3,712) = 22.632, *p* < 0.001. Planned contrasts of grade categories and enrollment showed that the older boys were much less likely to climb than the younger boys as shown by the interaction of the K through 2nd-grade category and the 3rd- through 6th-grade category: likelihood ratio χ^2^_1_ (*n* = 3,911) = 16.537, *p* < 0.001. In contrast for girls, the interaction of the K through 2nd-grade category and the 3rd- through 6th-grade category approached statistical significance: likelihood ratio χ^2^_1_ (*n* = 3,848) = 3.701, *p* = 0.054.

**Table 2.** Contingency Table of the Number of Children Climbing Playground Structures Except Rings and Swings. Values in Parentheses Indicate the Number of Children Enrolled in Each Grade Category not Climbing at the Time of Sampling, Assuming no School Absences.

	Grade Categories	
	K-2nd	3rd-6th
Boys	44 (1982)	12 (1817)
Girls	70 (1837)	47 (1777)

## Discussion

For children in K through 2nd and 3rd through 6th grades, the results showed that reliably higher percentages of girls climbed playground equipment in elementary school compared with boys. Consistent with the NEISS injury data showing a greater age-related decline in falling injuries in boys, the frequency of boys climbing was reliably lower for boys in 3rd through 6th grades compared with those in K through 2nd grades. Although girls also exhibited a grade-related decline in climbing frequency, this difference was almost statistically significant. More specifically with regard to the climbing injuries shown in Study 1, the lower percentage of injuries in older boys might simply reflect a reduction in climbing motivation rather than these boys displaying a developmentally greater climbing capability than older girls. However in the current study, we did not examine the participation of boys and girls in other playground activities, so we are limited in interpreting why older boys apparently lose interest in climbing. In a Canadian study, Wagner (1993) reported that twice as many first-grade children were observed on climbing structures than third-grade children. For both grade categories, more girls were observed climbing than boys and, according to Wagner (1993), this difference could reflect boys seeking more physically demanding activities than girls engendering greater injury risks (also see Ginsberg & Miller, 1982). Nevertheless, the aforementioned grade-related sex difference in climbing frequency might be limited to elementary school children rather than younger preschool children. For example, Williams and Beeson (1980) reported that preschool children showed no sex difference in the frequency of climbing during play periods.

An age-related change in risk assessment might play a role in the decline in climbing interest. Older boys have generally accumulated more minor playground injuries than would be expected to enhance their assessment of climbing risks. Using drawings presented to 6 to 11 year-old children showing children engaged in risky and safe bicycling, climbing, swinging, and sliding activities for rating their own injury risks along with those of peers, Morrongiello and Rennie (1998) reported that older boys showed a higher “optimism bias” in which they downplayed their risks of injury compared with those of older girls. Such a finding does not support our suggestion that children's assessment of experiencing potential injury while climbing influenced the decline in climbing motivation in older boys.

There are possible effects of sex stereotyping that emerge with age that would likely augment a disinclination to climb playground structures by older boys. According to Carter and McCloskey (1984), climbing in girls could be considered a “cross-gender” activity and children in their study reported that they would respond negatively to children expressing cross-sex-typed traits (also see Halliday & McNaughton, 1982). Nevertheless in a descriptive study of playground activities in 67 New England schools, climbing on jungle gyms was noted as a popular activity in girls with few boys observed climbing. Climbing in this gendered context was interpreted as a “girl's game” in which boys who were not playing team sports could still participate (see Boyle et al., 2003, p. 1332). Moreover when children were being chased, the girls were attracted to climbing structures as refuge because they tended to climb up or run around these structures whereas boys would run through groups of other children to avoid being caught.

The next study examined the construct that children can recognize that elevated promontories afford better viewing of the surrounding landscape. Using black silhouettes of four trees, Coss and Moore (2002) found that 3- to 5-year-old American, Israeli, and Japanese children selected taller than shorter trees as the best trees to “climb to see better.” If presumed ancestral niche partitioning due to historical differences in body size still influences climbing motivation to see better, we expected that girls would climb tall playground structures for this purpose more than boys.

## Study 3: Perching on Climbing Structures

During focal sampling of children climbing on playground structures during recess, it was noted that the girls appeared less active (more stationary) than boys. Such anecdotal observations suggested that girls might be using playground structures for viewing the activities of other children. As discussed above, other studies of playground activity (e.g., Reimers et al., 2018) and types of injuries (Farnsworth et al., 1998) suggest that boys are more physically active than girls. As such, we predicted that girls would exhibit more stationary behavior on the top of each climbing structure, a behavioral property we interpreted as perching for visual surveillance. Therefore, we selected a community park for study with small numbers of visitors to allow measurements of children climbing alone.

## Materials and Methods

### Participants

Twenty three boys (age 6.8, SD = 1.5 years) and 25 girls (age 7.02, SD = 2.1 years) were observed climbing three playground structures free of other climbers. This study was conducted in accordance with UC Davis IRB Human Subjects Protocol 91–243. Prior to sampling the children, their parents or guardians were approached at the study site and the rationale and methodology of the study were described on permission forms that requested information on the child's age. No ethnic information was requested and caregivers were informed that climbing measurements would be terminated immediately if requested. The study was conducted over a two-month period and each participant was sampled one time.

### Study Site

**Figure 4.**
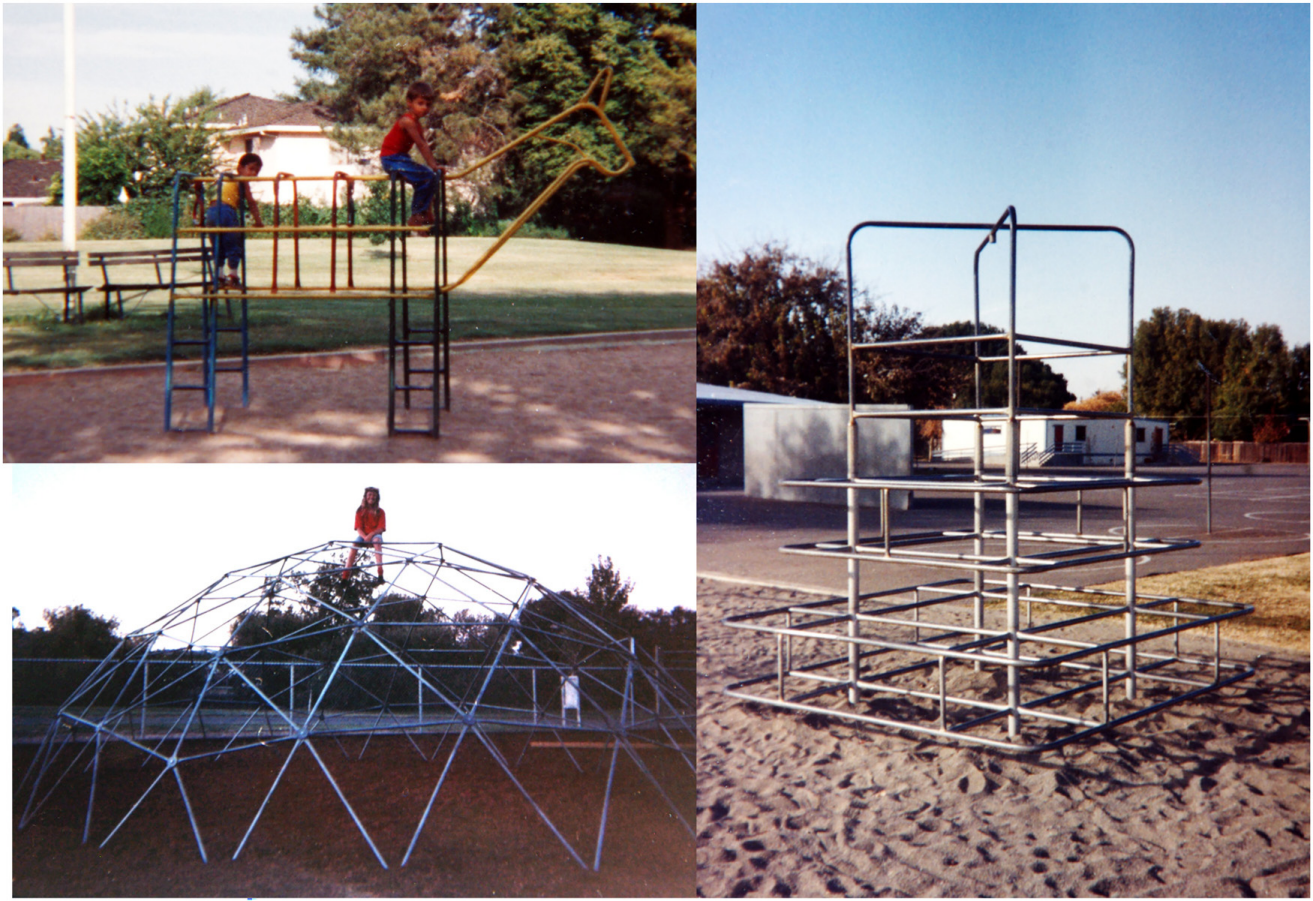
Children perching on three climbing structures in Ridgeview park, Fairfield, California.

Three playground structures of varying height were examined for perching behavior at Ridgeview Park, Fairfield, California. Variation in structure height affords different elevations for landscape viewing, including the activity of parents or guardians. The lowest climbing structure was a metal-tubed schematic horse (1.63 m height), the second tallest structure was a metal-tubed pyramid (2.86 m height). The tallest structure was a metal-tube geodesic dome (3.05 m height, 7.62 m dia.). The tops of these structures provided areas suitable for solitary perching (Figure 4).

### Behavioral Measure

The percentages of time climbing and perching on top of the three structures were measured using digital stopwatches. Since the duration of climbing varied among the children, it seemed appropriate to select a dependent variable that characterized the child's final climbing action on the highest accessible portion of each structure. As such, the percentage of time perching was derived as a proportion of the overall time of climbing alone and remaining stationary. This value was converted to an arcsine transformation for analysis by a two-factor (sex and 3 structures) analysis of variance (ANOVA).

## Results

**Figure 5.**
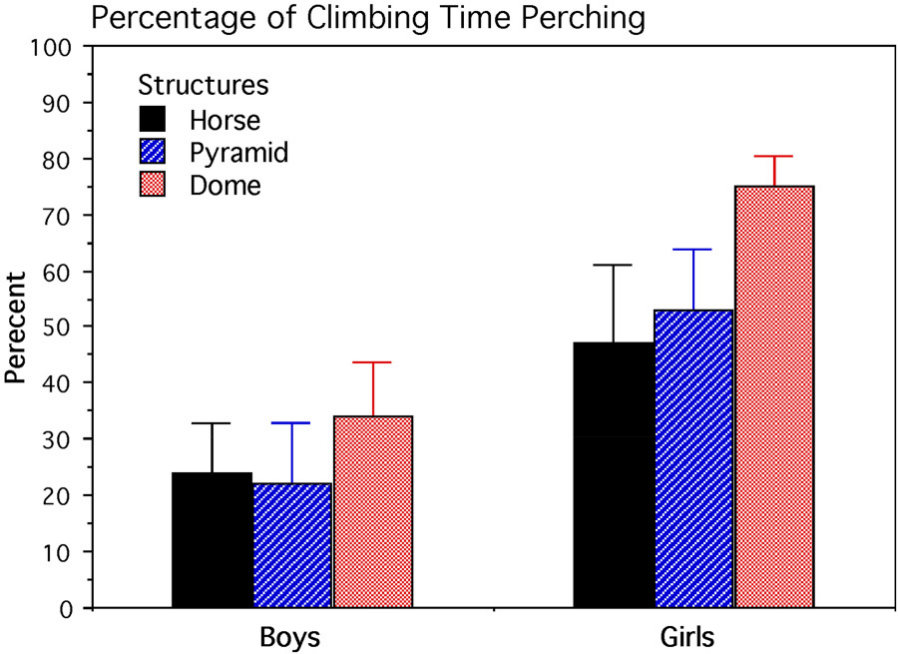
Differences in perching behavior as a function of overall climbing time. Averaged for climbing structure height, girls perched reliably longer than boys (*p* = 0.0004). Mean and standard-error values are shown.

Levene's Test for Homogeneity of Variances indicated a normal distribution of data. Averaged for structures, the main effect for sex was statistically significant, with girls perching for a longer percentage of time than boys, yielding a large standardized effect size (*F*_1, 42_ = 14.719, *p* = 0.0004, *d* = 1.1). The sources for this sex difference were analyzed further by examining the simple main effect for each climbing structure. For the shortest structure (horse), a test of simple main effect showed that boys and girls did not differ appreciably in perching percentages (*p* = 0.160). However for the two taller structures of similar height, tests of simple main effects indicated that the girls perched a significantly longer percentage of overall climbing time than boys did (Figure 5). The actual mean percentage values and 95% confidence intervals are as follows: pyramid, boys = 22.22 ± 16.51%, girls = 53.45 ± 21.12% (*F*_1, 42_ = 5.813, *p* = 0.020); dome, boys = 34.55 ± 18.93%, girls = 75.18 ± 11.13% (*F*_1, 42_ = 8.825, df = 1, 42, *p* = 0.005).

## Discussion

The findings of this study support our hypothesis that young girls would perch on climbing structures for longer percentages of climbing time than boys, notably when climbing taller structures. On these taller structures, girls perched more than twice as long as boys did in relation to their total time climbing each structure. Since the children sampled were not interviewed about where they were looking, we can only speculate about what park features attracted their attention. The tops of these playground structures afforded far-reaching views of lawns, trees, and walkways with occasional children on bicycles and adults walking their dogs. To reduce their vulnerability during Pliocene times, ancestral hominins would likely have used the elevated vantage point of trees for monitoring conspecifics and for detecting potential predators in a manner similar to the sentinel behavior of other primates (cf. Coss & Ramakrishnan, 2000; Enstam & Isbell, 2004; Horrocks & Hunte, 1986; McGraw & Bshary, 2002).

None of the three climbing structure appeared difficult to climb, two of which had metal bars arranged as ladders (see Figure 4). Ladder climbing in 7- to 9-year-old children has been studied previously with evidence that boys do climb faster than girls (Butcher, 1991), but there is no appreciable sex difference in climbing time in 4-year-old children (Plevnik et al., 2014). Our next experiment evaluated the visuomotor aspects of children inexperienced in climbing a rock wall in a climbing gym.

## Study 4: Indoor Rock Climbing

To approximate some of the limb actions and coordination in free-climbing trees without the inherent danger of falling (Gull et al., 2018; Jain et al., 2014), we selected a climbing gym as a study site for a descriptive study of sex differences in climbing action patterns. Consistent with this perspective, Carroll (2021) considers that rock climbing is the “natural analog” to tree climbing.

## Materials and Methods

### Participants

Twenty eight children 7 through 12 years of age were enrolled in a beginning indoor rock-climbing class at the Rocknasium gym, Davis, California, one of the oldest climbing gyms in the United States. Participants consisted of 15 boys, averaging 9.2 year of age, and 13 girls, averaging 9.4 years of age. The class focused on equipment, knot-tying, belaying, and climbing techniques, and met twice a week for two hours. All participants were inexperienced indoor rock-wall climbers, having never engaged in climbing in a gym using a belayer (another person who acts as an anchor for the climber as they scale a wall). Prior to video recording participant climbing behavior, the parents of these children received human-subjects permission forms for their signatures.

The class had five instructors and 10 climbing sessions were video recorded during two 5-week sessions (April, May, June) and (October, November, December, 2004). All 28 children used commercially available climbing gear and were secured to a rope and a belayer standing adjacent to the 4.88 m height vertical climbing wall. Since our research focus was multivariate motor-pattern relationships during belayed rock-wall climbing with participant selection of wall-climbing routes, rather than a standardized array of hand and footholds for stepping and reaching, ethnicity, height, weight, and body mass were not included as variables for analyses. Also, the irregularity of vertical and horizontal spacing of wall holds precluded studying gait-cycle phases (see McIntyre et al., 1982). This study was conducted in accordance with a renewal of UC Davis HRS Human Subjects Protocol 96-482R.

### Climbing Measures

**Figure 6.**
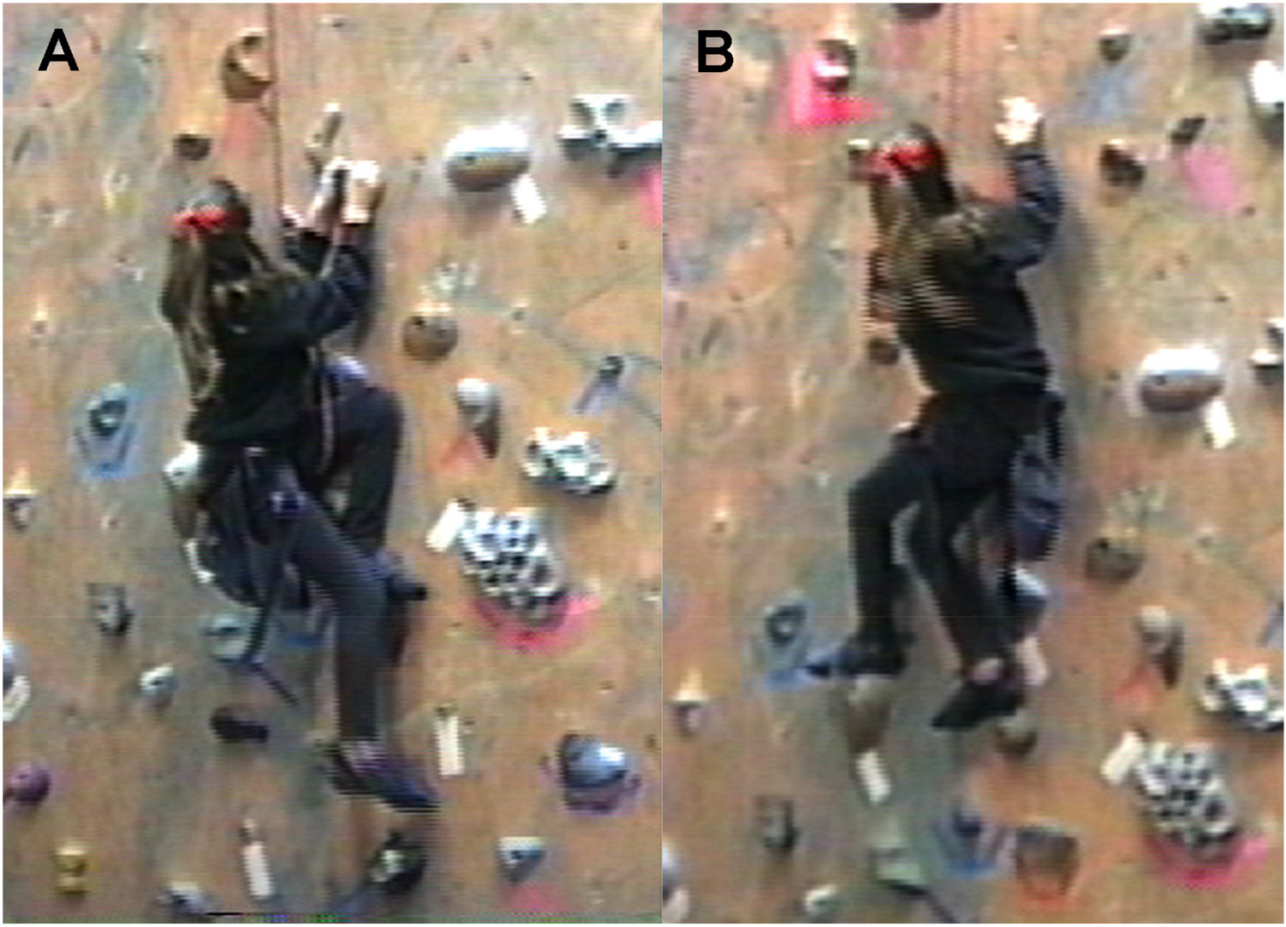
Video still-frames of a girl in a beginner rock-wall climbing class exhibiting two-handed clasping of a support (A) and crossover stepping by twisting the body (B).

The frequency of specific climbing action-patterns on the climbing wall was determined by counting the actions as percentages of the total number of reaches and steps taken while the child ascended the wall. Climbing behavior was video recorded using a Panasonic VHS camcorder model AG-185U. The camera location was elevated and unobtrusive, requiring the use of close-up telephoto imaging for behavioral quantification of body profiles. Only one wall-climbing event was recorded for each child. After initial analysis of the video recordings of wall climbing, the following four behaviors were selected for quantification as a percentage of reaches and steps: 1) the frequency of two-handed clasping of the same hold to aid in pulling oneself up the wall to the next foot support (Figure 6A); 2) the frequency of crossover stepping by twisting the body (Figure 6B); 3) the frequency of stepping with one foot towards another sought-after foothold while dragging the other foot up the wall with no apparent foothold in mind; 4) the frequency of looking (via head orientation) to position a foot on a hold. These four behaviors were decoded by two investigators sitting side-by-side using video frame-by-frame analyses with repeated playbacks for measurement accuracy. Only one behavior was quantified at a time.

## Results

**Figure 7.**
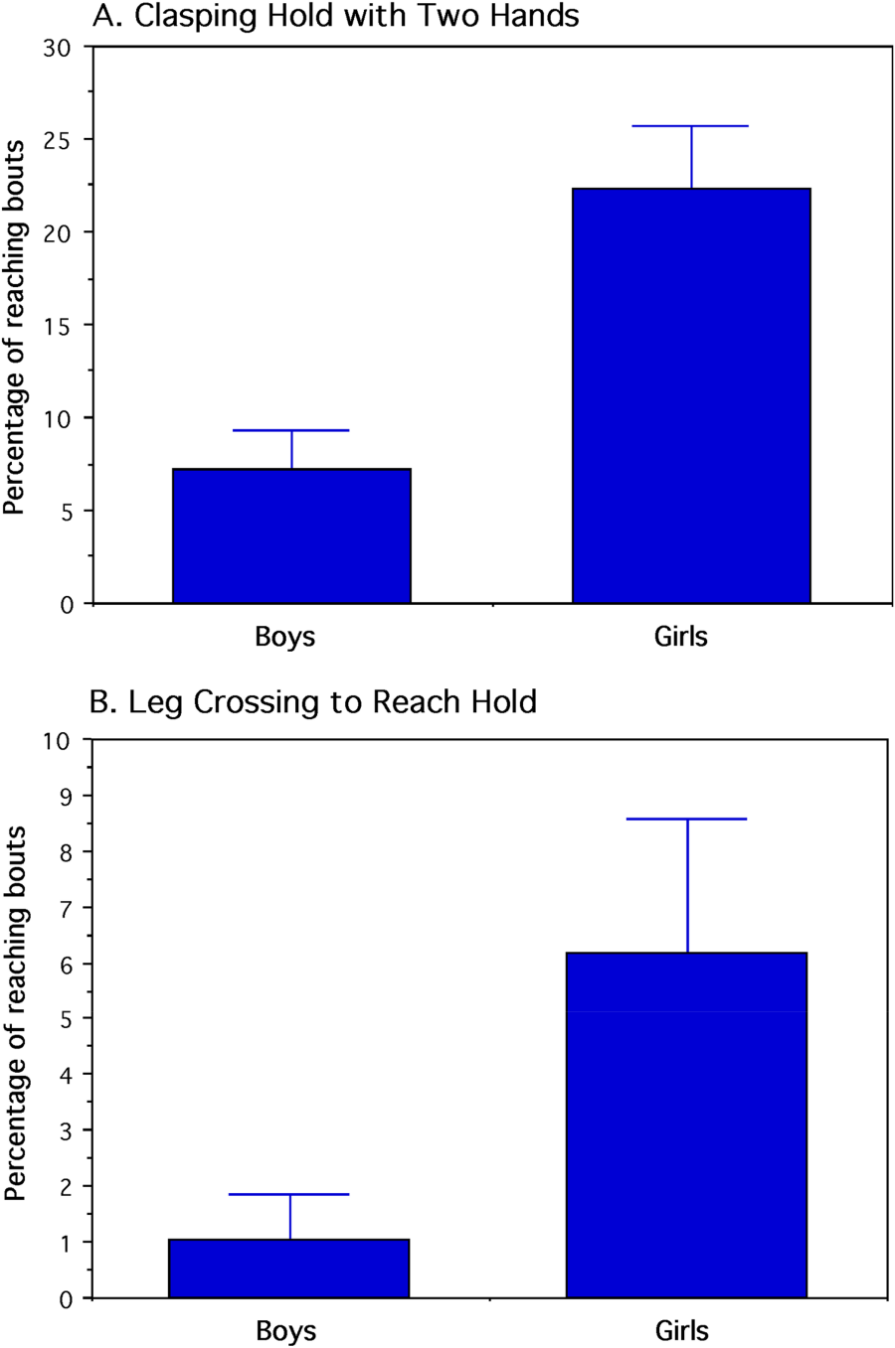
Percentage of hold-reaching bouts in which two-handed clasping of a hold was exhibited (A) and the percentage of bouts in which leg crossing occurred to reach a hold with a foot (B). For both behaviors, the girls differed reliably from the boys (*p* < 0.05). mean and standard-error values are shown.

**Figure 8.**
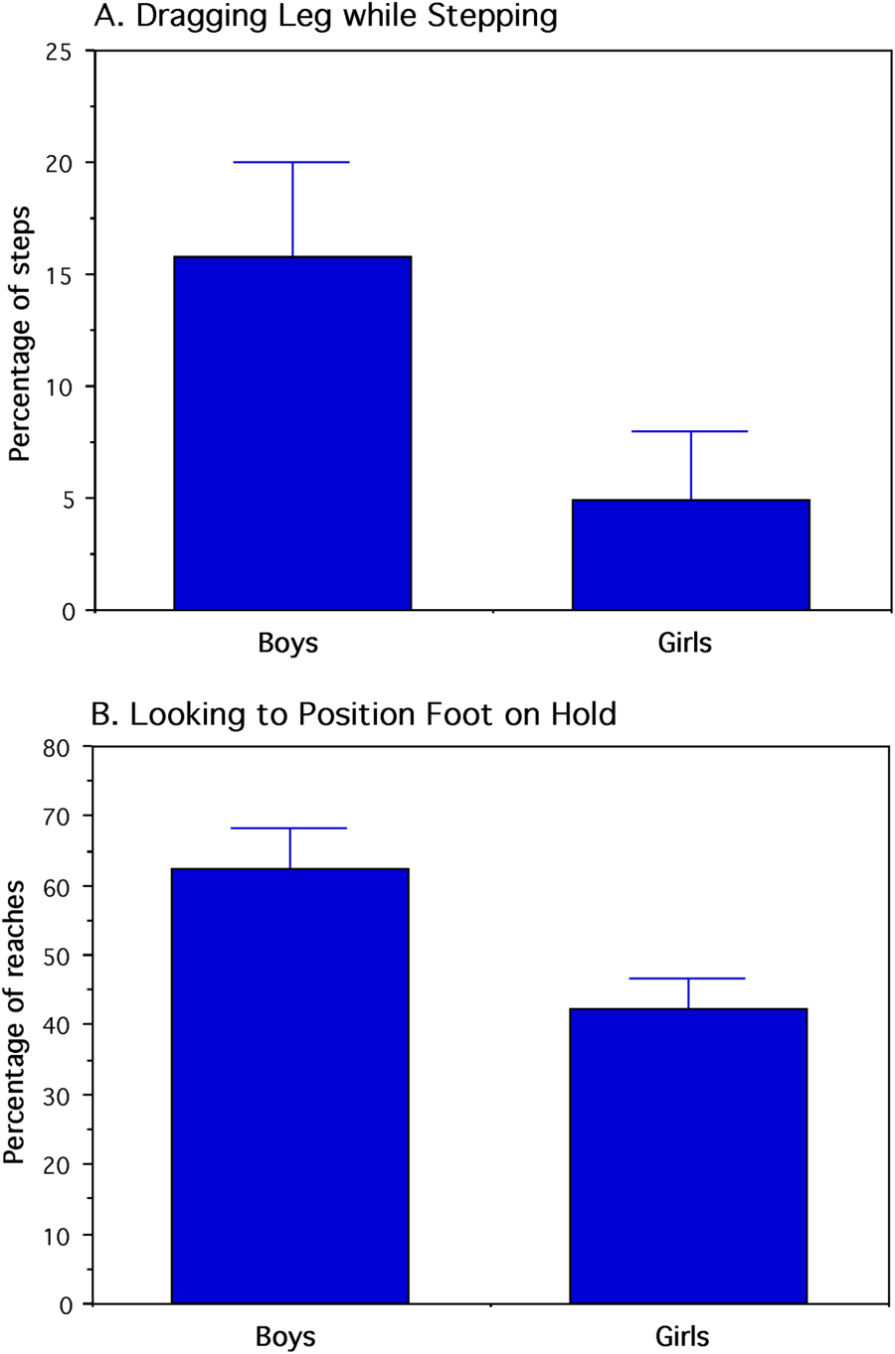
Percentage of stepping bouts in which one leg was dragged while the other foot stepped upwards (A) and while looking in the direction of where to position a foot on a hold (B). For both behaviors, the girls differed reliably from the boys (*p* < 0.05). Mean and standard-error values are shown.

The four behavioral measures in percentages were transformed into arcsine values for discriminant function analysis that classified each child by sex. To test the assumption of multivariate normality for a MANOVA of the four dependent variables, Box's *M* test showed that the variance-covariance matrices were homogeneous. Following this test, a single-factor MANOVA indicated a significant sex difference (*Pillai-Bartlett Trace*_4,23_ = 0.463, *p* = 0.005. All four univariate analyses were statistically significant with large standardized effect sizes. Actual mean percentages with standard errors are shown in Figures 7 and 8 and the actual mean percentages with 95% confidence intervals are reported here: 1) two-handed clasping of a hold: boys = 7.3 ± 4.1%; girls = 22.4 ± 6.7% (*F*_1,26_ = 11.060, *p* = 0.003, *d* = 1.3). 2) crossover stepping by twisting the body: boys = 1.1 ± 2%; girls = 6.2 ± 4.7% (*F*_1,26_ = 4.654, *p* = 0.040, *d* = 0.85). 3) dragging foot while stepping: boys = 15.9 ± 8.2%; girls = 5.0 ± 6.1% (*F*_1,26_ = 5.268, *p* = 0.030, *d* = 0.90). 4) looking to position the foot on a hold: boys = 62.8 ± 6.1%; girls = 42.7 ± 8.2% (*F*_1,26_ = 6.007, *p* = 0.021, *d* = 0.96). The classification matrix with a 0.5 probability showed that 13 boys (86.67%) and 11 girls (84.62%) were correctly classified by their sex. Among the dependent variables contributing to group classification, leg dragging yielded the smallest standardize coefficient for canonical variables.

## Discussion

Among rock-wall climbing beginners, discriminant function analysis documented at the multivariate level that girls differed from boys in the 4 action patterns of wall climbing selected for study. The first dependent variable that was conspicuously exhibited by the girls was a two-handed clasp of a hold. It was not apparent that this action was immediately beneficial for positioning the body for the next climbing action. Due to their cylindrical properties, two-handed grasping of tree branches can be observed in children climbing trees (pers. obser.), a property also revealed by branch polishing with repeated grasping. Thus, two-handed clasping of holds during wall climbing might reflect a more intuitive climbing inclination in girls.

The second dependent variable was crossover stepping while twisting the body. This climbing action seemed to show the lateral balancing technique previously described by Testa and Debu (1997) as postural adjustments. Even though the body was being twisted, the execution of the climbing action kept the lateral balance needed in order to achieve the desired ascent up the wall. A sex difference in twisting the body might also reflect the greater hip rotation found in girls and retained in women (cf. Svenningsen et al., 1989, p. 96; Bussey et al., 2009; Beneck et al., 2018).

The third and fourth variables chosen both worked in accordance with each other. The variables chosen were, steps with leg dragging and steps while looking at the feet. While examining the videos, we noticed that some of the children seemed to be memorizing their steps during their ascent up the wall. This enabled them to make three or four steps without looking at their feet and where the next foot-hold was located. So it appears that beginner climbers were either mapping their route before or during their ascent. According to Rocknasium instructor Danica Fitts (pers. com., Jan. 2004), hand-hold visualization is taught to competitive rock climbers. They are taught to memorize where their hand-holds are located so that they can then use them for their feet. Therefore, the lack of looking at their feet during their placement on holds by beginning climbers seemed to indicate an advanced, advantageous climbing technique as evidenced by the girls. In contrast, dragging one leg while stepping with the other appeared subjectively to be both inefficient and less coordinated. It seems reasonable to assert here that children who were not memorizing their next climbing steps would likely require visual monitoring of their foot placements on holds. Research on route learning in children has shown that boys typically perform better than girls, with boys appearing to depend more on Euclidean cues for navigation (e.g., Beilstein & Wilson, 2000; Merrill et al., 2016). However, it seems that girls are more effective learners of landmark features as navigation guides (Gibbs and Wilson, 1999; Bocchi et al., 2020). Although physically close during indoor rock-wall climbing, the spatial array of potential holds would presumably have similar landmark properties useful for selecting climbing routes (see Pezzulo et al., 2010; Whitaker et al., 2020). Although speculative for routine tree climbing by human ancestors, the three-dimensional array of branch locations as potential holds would probably have been perceived as climbing navigation landmarks.

Leg dragging occurred when a step was made with one foot and then the child would look up to their next hand-hold. During this time the child would be dragging his or her other foot up the wall with no apparent destination for it in mind. As the hand-hold was located and grasped, the child would then scan for another support for the previously dragged foot. This inefficient climbing action was not the same as the climbing technique used by climbers known as “flagging” (Goddard & Neumann, 1993), a technique used by well-trained climbers not an easily acquired according to Rocknasium instructor Rebecca Norvelle (pers. com., Feb. 2004).

## General Discussion

The primary rationale for conducting the current auxiliary studies of sex differences in children's climbing motivation and action patterns was to examine additional behaviors that might be relicts from a time frame in which sexual-size dimorphism constrained arboreal behavior (see Coss & Charles, 2004, for discussion of progressive-science). Evolutionary persistence of behavior under prolonged relaxed selection requires extensive pleiotropic interactions within gene regulatory networks shaping phenotypic expression (i.e., Sinha et al., 2020; Zhang, 2023). Such preservation of specialized gene regulatory networks organizing brain development (Li et al., 2018; Lodato & Arlotta, 2015; Silbereis et al., 2016, p. 249) and variability in behavioral expression would likely be buffered from undergoing marked reorganization under relaxed selection due to their intertwining connectedness with other networks still experiencing natural selection. As discussed previously, climbing indubitably has multifunctional properties involving extensive visuomotor coordination useful in a variety of non-arboreal situations.

Precocious expression of climbing activity in young children with undeveloped brains arguably reflects the early installation of neural circuits originally useful later in development for promoting the reproductive fitness of adult ancestors via successful arboreal foraging and predator evasion. Based on the manner in which neurons grow dendrites, such neural circuits for behavioral precocity would need to be installed on the robust properties of early neuronal outgrowth less subject to experiential remodeling (e.g., Volkmar & Greenough, 1972).

With regard to the aforementioned discussion of developmental hypermorphosis within the human lineage, the accelerated decline in climbing motivation in boys after age 6 might reflect the ancestral maturational state of early hominins with faster growth; some forms of behavioral development retain their ancestral developmental timing because they are deeply canalized (cf. De Beer, 1958; Levis and Pfennig, 2020). Within this context, one can envision that more mature male australopiths were endangered when they attempted to climb on the same weight-bearing branches used at a younger age or used by smaller females to forage (for gorillas, see Remis, 1995). Although our primary explanation for the decline in climbing in boys and girls is the attraction to alternative social and sports-related playground activities, it is still reasonable to consider that these sexually divergent developmental changes in climbing motivation might have an evolutionary origin (for further discussion, see Carroll, 2021).

Another attribute that motivates children's climbing appears to be visual surveillance facilitated by jungle gyms of older designs with views unencumbered by walls and safety railings that restrict widespread scanning of the surroundings. Based on the sexual-dinichism construct, we predicted that girls would perch on these structures to look around for longer periods than would boys. Other than for surveillance from an elevated perspective, the physical inactivity of solitary perching suggests that a positive sense of well-being can emerge from understanding that being elevated affords protection (for discussion of window views of urban landscapes, see Wang et al., 2019). In a historically hostile environment with terrestrial and arboreal predators, australopith females high up in trees might have recognized this safe arboreal affordance more than much-heavier males restricted in climbing proficiency (Harcourt-Smith, 2015, p. 13; Stamos and Alemseged, 2023).

During rock-wall climbing using a belayer for safety and to engender climbing confidence, the boys looked more often in the direction of their feet to position them on holds. Climbing an unfamiliar rock-wall by beginners clearly requires greater spatial planning and motor coordination than simply relying on the automaticity of ladder stepping derived from previous ladder-climbing experiences on playground slides. In our study conducted at the Rocknasium climbing gym in Davis, California, beginner girls appeared to exhibit more efficient rock-wall climbing than the boys did, a property that might characterize an evolutionarily based retention of climbing aptitude as a behavioral relict of ancestral sexual-size dimorphism.

Though incomplete, our research findings lend support to our conjecture that a sex difference in children's desire to climb reflects the forward-causal process of evolutionary persistence of behavior originating deep within the human lineage in which sexual-size dimorphism constrained climbing behavior. Due to contemporary changes in playground climbing structures based on safety concerns, future studies of sex differences might focus on standardized sets of climbing challenges as afforded by climbing gyms coupled with survey instruments to assess children's attitudes about climbing. Any sex differences in motor skills that emerge might be correlated with prenatal exposure to testosterone (see Lombardo et al., 2018).
